# The Vaccination Papers of Dr. Leverson

**Published:** 1898-06

**Authors:** 


### THE VACCINATION PAPERS OF DR. LEVERSON.

In the March number the Editor of this journal requested the opinions of the profession upon the vaccination papers which have been continued in this journal during a year and a half past.

A number of replies have been received, from which the following have been selected as indicating the tendency of opinion in the profession.—EDITOR.

---

